# Network pharmacology and experimental validation to explore the potential mechanism of Sanjie Zhentong Capsule in endometriosis treatment

**DOI:** 10.3389/fendo.2023.1110995

**Published:** 2023-02-03

**Authors:** Ruoyi Guo, Zhihui Yi, Yun Wang, Li Wang

**Affiliations:** Department of Gynecology, Obstetrics and Gynecology Hospital, Fudan University, Shanghai, China

**Keywords:** Sanjie Zhentong Capsule, endometriosis, network pharmacology, molecular docking, steroid metabolism, apoptosis, ROS

## Abstract

**Purpose:**

Sanjie Zhentong Capsule (SZC) is gradually becoming widely used in the treatment of endometriosis (EMs) and has demonstrated an excellent curative effect in the clinic. However, the active components and mechanisms of Sanjie Zhentong Capsule (SZC) in the treatment of endometriosis (EMs) remain unclear, and further research is needed to explore the effects of Sanjie Zhentong Capsule (SZC).

**Materials and methods:**

First, a drug target database of Sanjie Zhentong capsule (SZC) was established by consulting the TCMSP database and related literature. An endometriosis (EMs) disease target database was then established by consulting the GeneCards, OMIM and Drug Bank databases. The overlapping genes of SZC and EMs were determined, and protein-protein interactions (PPIs), gene ontology (GO) and Kyoto Gene and Genome Encyclopedia (KEGG) analyses were performed to predict the potential therapeutic mechanisms. Molecular docking was used to observe whether the key active ingredients and targets predicted by network pharmacology had good binding energy. Finally, *in vitro* experiments such as CCK-8, flow cytometry and RT-PCR assays were carried out to preliminarily verify the potential mechanisms.

**Results:**

Through the construction of a pharmacological network, we identified a total of 28 active components in SZC and 52 potential therapeutic targets. According to GO and KEGG enrichment analyses, the effects of SZC treatment may be related to oxidative stress, steroid metabolism, apoptosis and proliferation. We also experimentally confirmed that SZC can regulate the expression of steroid hormone biosynthesis-related genes, inhibit ectopic endometrial stromal cell (EESC) proliferation and oxidative stress, and promote apoptosis.

**Conclusion:**

This study explored the potential mechanism of SZC in the treatment of EMs through network pharmacology and experiments, providing a basis for further future research on SZC in the treatment of EMs.

## 1 Introduction

Endometriosis (EMs), a chronic gynecological inflammatory disease associated with dysmenorrhea and infertility, is defined as endometrial implantation outside the uterus, often in the pelvis, in forms such as superficial peritoneal lesions, ovarian EMs cysts and deep infiltrating EMs. EMs lesions are benign in essence, but they are characterized by a propensity for recurrence and strong invasiveness, similar to malignant tumors. The incidence of this disease in women of childbearing age is up to 10% (1) and has been increasing annually. The main symptoms are progressively worsening dysmenorrhea, chronic pelvic pain, infertility and discomfort during sexual intercourse (2).

The treatments for EMs include mainly surgery and medical treatment (3). Laparoscopy is the gold standard for the diagnosis and classification of endometriosis, aims to reduce endometriosis-associated pain and restore normal anatomy (4), but it is associated with risks of recurrence and repeat surgery. Medical treatments mainly include hormone therapy and NSAIDs, which can treat EMs by inhibiting ovarian funtion, suppressing menstruation, and resulting in endometrial atrophy, relieving symptoms (5). However, there are some side effects, such as irregular menstrual bleeding, fluid retention, gastrointestinal reactions, and are not suitable for women who is seeking a pregnancy, so it is important to develop new treatment strategies and drugs for EMs.

In traditional Chinese medicine (TCM), EMs is considered a form of blood stasis in women, and the symptoms of EMs are classified as “dysmenorrhea”, “gynecologic abdominal lumps” and “infertility”( 6). The application of TCM in the medical treatment of EMs is becoming increasingly common, and have shown its remarkable therapeutic effect in the prevention and treatment of EMs (7), can be an alternative treatment of EMs in China owing to its well efficacy and low toxicity (8).

Sanjie Zhentong Capsule (SZC) is a traditional Chinese medicine consisting of the original powder of four natural plants products, Resina Draconis, *Panax notoginseng*, Fritillariae Thunbergii Bulbus, and Coicis Semen. SZC softens and dissipates knots, eliminates blood stasis and relieves pain (9–15). In recent years, SZC has demonstrated excellent therapeutic effecacy in the clinical treatment of severe adenomyosis and EMs, as it can relieve dysmenorrhea and effectively inhibit the growth of lesions (9), and SZC is widely used in the clinic because of its few side effects and good safety (12). Our previous study showed that SZC was more effective than a gonadotropin−releasing hormone analog (GnRH-α) or oral contraceptives (OCs) in relieving pain and improved quality of life (QoL) with fewer adverse events in patients with moderate−to−severe EMs (16). Other studies showed that SZC could significantly reduce the lesion size in an EMs rat model, affect the biological processes of inflammation and angiogenesis by inhibiting the expression of VEGF and TNF-α (11) and exhibit efficacy superior to that of Danazol in reducing the levels of prostaglandin E2 (PGE2) and promoting apoptosis (17).

However, due to the diverse etiology of EMs and the lack of studies on the mechanisms of SZC, the active ingredients and potential mechanisms of SZC in the treatment of EMs should be studied to optimize the use of SZC in clinical drug regimens.

The effects of TCMs, especially compound preparations, often involve multiple components, targets, and pathways. Therefore, it is difficult to study the mechanisms of TCMs. In recent years, the emergence of network pharmacology has relieved the limitations of “single component–single target–single pathway “ research in the TCM field to some extent.

**Figure 1:**
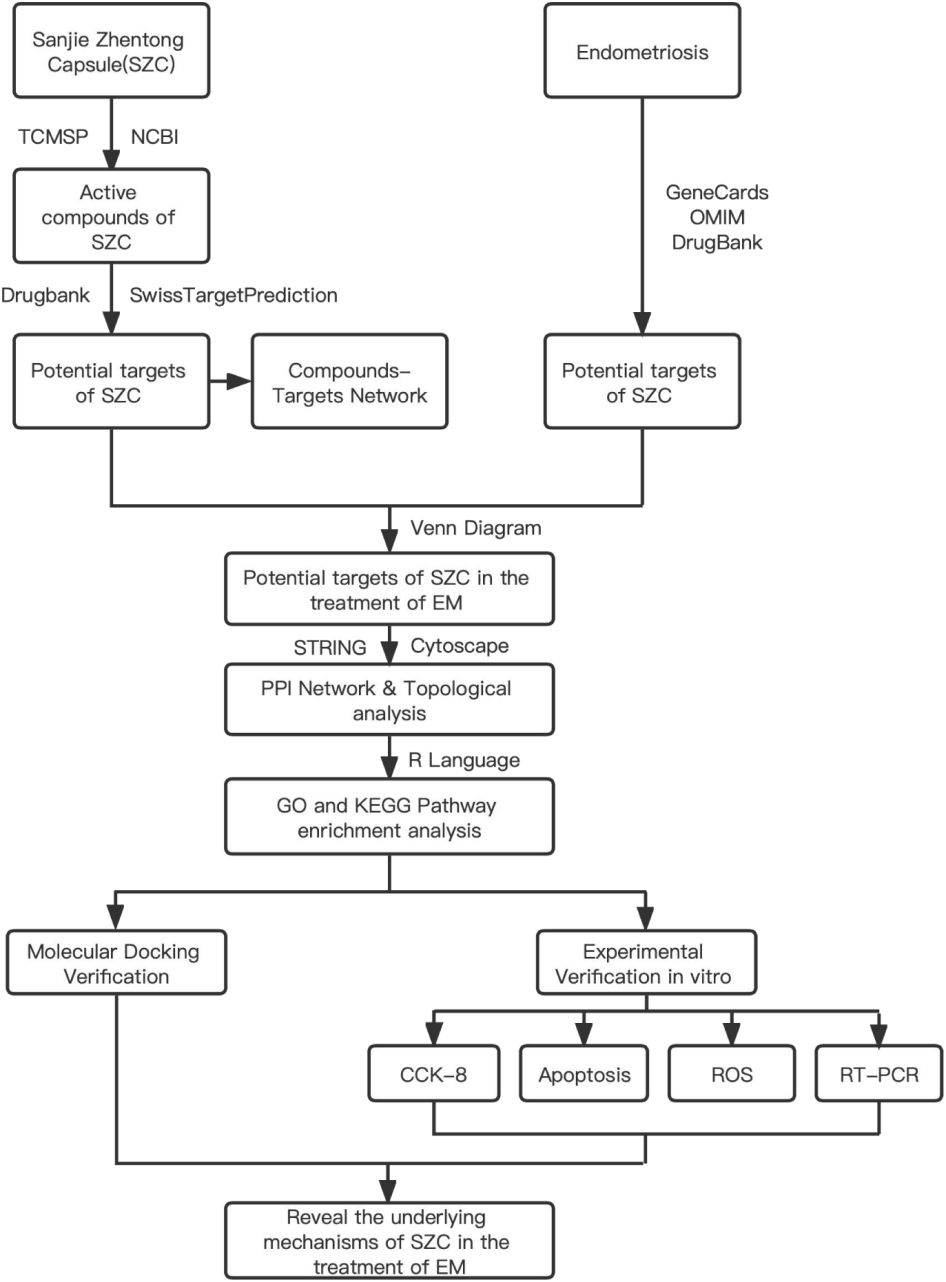
Flow diagram of the study of the study of SZC in Ems Treatment.

The concept of network pharmacology was first proposed in 2007 by Hopkins (18), a pharmacologist at the University of Dundee, UK. This field integrates pharmacology, bioinformatics and computer technology and in volves constructing a visualization network consisting of multiple components, targets, and pathways. It is used to predict potential active components of TCMs and related targets in order to understand the relationship between TCMs and diseases and to clarify the potential mechanisms of action of TCMs (19–21). One study used network pharmacology to verify that SZC can be used to treat adenomyosis through its anti-inflammatory activity and revealed that 30 potential active ingredients and 28 potential core targets may interact through 4 key pathways (22). However, the mechanism of SZC in the treatment of EMs remains unclear. Therefore, we systematically analyzed this mechanism through network pharmacology and related validation experiments to explore the main active components of SZC and the potential therapeutic mechanism of this TCM. The workflow is shown in **Figure 1**.

## 2 Materials and methods

### 2.1 Construction of the SZC compound database

To elucidate the possible chemical compositions of SZC, we obtained information on the components of SZC through the TCMSP database and established a SZC compound database. Resina Draconis, one of the main components of SZC, is not included in the TCM-related databases. We obtained its compounds and structural formula from the literature in PubMed and the China National Knowledge (CNKI) database and used PubChem to obtain the 3D structure. For some components, no relevant structural formula was available in PubChem, so we used ChemDraw to draw the structural formulas for subsequent screening of potential active ingredients.

### 2.2 Potential active component screening

With the established SZC database, we further screened the potential active components of SZC with good metabolism according to the absorption, distribution, metabolism and excretion (ADME) characteristics of the drug. For the compounds that were listed in the TCMSP database, we set a drug oral bioavailability (OB) ≥ 30% and druglikeness (DL) ≥ 0.18 as the screening criteria. Since Resina Draconis is not contained in the various drug databases, we obtained the chemical structures of related compounds by referring to the literature. Potential active ingredients were screened using SwissADME (http://www.swissadme.ch/) with the following criteria: (1) “high” gastrointestinal (GI) absorption and (2) “Yes” for at least 2 of the 5 DL characteristics.

### 2.3 SZC target prediction

To obtain the target genes corresponding to the potential active ingredient of SZC, its 3D structures were imported into SwissTargetPrediction (www.swisstargetprediction.ch/), the species was set to *Homo sapiens*, and the probability was set to >0.2.

### 2.4 Construction of the EMs therapeutic target database

To obtain potential therapeutic targets for SZC in the treatment of EMs, we first obtained the relevant targets in EMs from the GeneCards Database (www.genecards.org/), OMIM database (www.omim.org/) and DrugBank database (go.drugbank.com/) with the keyword “endometriosis”. These repositories are all human gene databases that provide comprehensive information on predicted human genes and are often applied in network pharmacology. After obtaining the targets corresponding to the potential active ingredients of SZC and the EMs, the overlapping targets were obtained by drawing a Venn diagram online (bioinfogp.cnb.csic.es/tools/venny/index.html) and the EMs therapeutic target database was established.

### 2.5 PPI analysis

Protein−protein interaction (PPI) analysis was performed on the therapeutic targets to screen out key therapeutic targets, The therapeutic targets obtained previously were imported into the STRING database (cn.string-db.org/), and the organism was set as *Homo sapiens*, and the confidence was set to ≥0.4, to obtain a protein–protein interaction (PPI) network. Cytoscape v3.9.0 software was used for topological analysis. In order to further screen the key targets, two parameters, “degree” and “betweenness centrality” (BC), were used in the topological analysis to evaluate PPIs that were positively correlated with the importance of the target in the SZC mechanism for EMs treatment.

### 2.6 GO and KEGG enrichment analyses

To further elucidate the therapeutic mechanism of SZC in EMs, we explored the biological functions of the drug and the possible pathways involved *via* Gene Ontology (GO) and Kyoto Encyclopedia of Genes and Genomes (KEGG) pathway enrichment analyses. GO analysis enables multifaceted description of the functions of a gene in three aspects: molecular function (MF), biological process (BP) and cellular component (CC). The KEGG database integrates genetic information for systematically analysis and further elucidation of gene functions, covering biological functions, pathways and other aspects. We used R language packages (including ‘ClusterProfiler’, ‘BiocManager’, ‘org.Hs.eg.Db’, ‘GOplot’, ‘RSQLite’, ‘colorspace’, ‘stringi’, ‘DOSE’, and ‘pathview’) to perform GO and KEGG pathway enrichment analyses of the previously predicted therapeutic targets. We visualized the results with a bubble chart (p<0.05) to further understand the expression of potential target proteins and the enrichment of the differentially expressed genes in biological functions and pathways.

### 2.7 Molecular docking study

We performed molecular docking of the key active ingredients and therapeutic targets predicted by network pharmacology for preliminary validation. Molecular docking is an important technique for structure-based drug design and screening in which the interactions between ligand molecules and receptor molecules are investigated and their affinities and binding modes are predicted, which are commonly used in drug research. After we obtained the crucial predicted targets of SZC for the treatment of EMs through PPI network analysis, we further verified them through molecular docking. The structures of the following crucial target proteins for SZC therapy were taken from the PDB database (https://www.rcsb.org/) and UniProt (www.uniprot.org/): matrix metallopeptidase 9 (MMP9; 6ESM), Estrogen Receptor 1(ESR2; 3OLL), cytochrome P450 (CYP) family 19 subfamily A member 1 (CYP19A1; 3S79), androgen receptor (AR; 5V8Q), AKT serine/threonine kinase 1 (AKT1; 1UNR), estrogen receptor 1 (ESR1; 6VIG), SRC proto-oncogene, non-receptor tyrosine (SRC; 1FMK), prostaglandin-endoperoxide synthase 2 (PTGS2; 5F19), CYP family 3 subfamily A member 4 (CYP3A4; 5VCC), epidermal growth factor receptor (EGFR; 5HG8), and AKR1C3 (1ZQ5). The software program AutoDock 1.5.6 was used to perform molecular docking between target proteins and active ingredients, and the binding energy from the molecular docking experiments was used as a docking score to assess the protein−ligand binding potential. Of the targets and ligands, combinations with a selection value of ≤-5 were considered to have moderate-to-tight binding potential. The smaller the binding energy is, the better the molecules bind.

### 2.8 Reagents

7,4’-Dihydroxyflavone (HPLC≥ 98%, MedChemExpress), kumatakenin (HPLC≥ 98%, MedChemExpress), quercetin (HPLC≥ 98%, MedChemExpress), liquiritigenin (HPLC≥ 98%, MedChemExpress), and SZC (Kanion Pharmaceutical Co., Ltd., Jiangsu, China). The obtained reagents were dissolved in DMSO.

### 2.9 Sampling

The protocol used in the present study was approved by the Human Research Ethics Committee of the Obstetrics and Gynecology Hospital, Fudan University, and all participants signed informed consent forms in compliance with the code of ethics set by the World Medical Association (The Declaration of Helsinki). We collected 20 ectopic endometrial specimens from women aged 20-45 years with ovarian EMs at the Obstetrics and Gynecology Hospital of Fudan University. None of these patients received relevant treatment in the three months prior to surgery. All cases were confirmed by pathology. Patient medical records were obtained through face-to-face interviews. The EMs specimens collected in this study were in the cell proliferation stage, and tissue specimens assessed as stage I-II according to the American Society for Reproductive Medicine (ASMR) criteria were collected for research. Approval was granted by the Ethics Committee of the Institutional Review Board Ethics Committee of the Obstetrics and Gynecology Hospital of Fudan University (2019-103).

### 2.10 Ectopic endometrial stromal cell isolation and culture

Human ectopic endometrial stromal cells (EESCs) were routinely isolated by previously described methods (23). Endometriotic tissue was washed with sterile PBS, cut into 1-3-mm pieces, treated with 1 mg/ml type IV collagenase (0.1%; Sigma−Aldrich; Merck KGaA; cat. No. C5138-1) containing 100 U/ml penicillin and 100 mg/ml streptomycin and digested with constant stirring for 30-45 min at 37°C. The EESCs were then filtered, the cell suspension was centrifuged at 1,200 rpm for 6 minutes at 4°C, and the supernatant was discarded. The obtained cells were mixed with DMEM (HyClone; GE Healthcare) containing 10% FBS (Gibco; Thermo Fisher Scientific, Inc.) and 1% penicillin−streptomycin and cultured in a 5% CO_2_ incubator at 37°C. Immunohistochemistry was performed on isolated cells. When >98% of cells were stained vimentin-positive, and <2% were stained CK-positive, the isolation and purification of EESCs were considered successful for further experiments.

### 2.11 Cell viability assay

The viability of EESCs following treatment with SZC and its components was assessed by the Cell Counting Kit-8 (CCK-8) assay. Cells were seeded in 96-well plates; treated with quercetin (0, 1, 2, 5, 10, 20, and 50 µM), 7,4’-dihydroxyflavone (0, 1, 2, 5, 10, 20, 50, 100 µM), liquiritigenin (0, 1, 2, 5, 10, 20, 50, 100 µM), kumatakenin (0, 1, 2, 5, 10, 20, 50 µM) and SZC (0, 1, 2, 5, 10, 20, 50, 100, 200 µg/ml); and incubated at 37°C for 24 hours. After discarding the medium, the cells were incubated with 10 μl of CCK-8 reagent (Beyotime Biotechnology, China) per well for 30 min at 37°C. The OD value of each well at 450 nm was assessed with a microplate reader (Bio-Rad). Subsequent apoptosis and ROS assays to test the effects of drug treatment concentrations were carried out based on the CCK-8 assay results.

### 2.12 Flow cytometry analysis of apoptosis

An Annexin V-FITC/Propidium Iodide (PI) Apoptosis Detection Kit (Beyotime Biotechnology, China) was used to evaluate the apoptosis of EESCs treated with SZC and related compounds. Cells were seeded in 6-well plates, treated with quercetin (20 µM), 7,4’-dihydroxyflavone group (20 µM), liquiritigenin group (20 µM), kumatakenin group (20 µM) and SZC group (200 µg/ml), and incubated at 37°C for 24 hours. Then, the cells were washed with cold phosphate-buffered saline (PBS) and digested with a trypsin-EDTA solution. The cells were resuspended in ice-cold PBS for cell counting and diluted with 195 μl of Annexin V-FITC binding buffer. Subsequently, 5 μl of Annexin V-FITC and 10 μl of PI were added, and then the cells were mixed well at room temperature and incubated for 15 minutes without light. The proportion of apoptotic cells was detected with a Beckman Coulter flow cytometer.

### 2.13 RT-PCR analysis

RT−PCR was used to validate the potential therapeutic targets which predicted by network pharmacology. EESCs were seeded in 6-well plates and divided into control group and SZC group, The SZC group was treated with 200 µg/ml SZC and incubated at 37°C for 24 h. Total RNA was extracted form the EESCs using the TRIzol^®^ reagent (Invitrogen, 15596018). The RNA was reverse-transcribed into cDNA utilizing a Prime Script RT Reagent Kit (Takara, RR036A). RT−PCR was performed using SYBR Premix Ex Taq (Takara, RR820A), and the results were analyzed using a ABI Prism 7900 Fast Sequence Detection System (Thermo Fisher Scientific). Each sample was analyzed in three replicate wells, and the fold change in the transcriptional expression of the above genes was calculated using the 2−ΔΔCt method. The relative mRNA expression levels were normalized to those of ACTB. The primer sequences of these genes are provided in **Supplementary Table S1**.

### 2.14 Measurement of intracellular ROS levels

The intracellular reactive oxygen species (ROS) levels were measured using a Reactive Oxygen Species Assay Kit (Beyotime Biotechnology, China), DCFH-DA was diluted to 10 μM in serum-free medium and added to cells treated with SZC and related compounds (at the same concentration as in apoptosis), which were incubated at 37°C for 20 min and washed with serum-free cell culture medium. After this, the mean fluorescence intensity of the cells was detected with a Beckman Coulter flow cytometer.

### 2.15 Statistical analysis

Data analysis was performed with GraphPad Prism v9.3.1. All results are expressed as the mean ± SD and were assessed using one-way ANOVA test and an unpaired t-test, with p<0.05 indicating statistical significance.

## 3 Results

### 3.1 Potential components and targets of SZC

By consulting the TCMSP database and related literature, we screened out a total of 40 bioactive components of SZC according to their ADME characteristics (**Supplementary Table S2, Supplementary Table S3**), mainly flavonoids, and phytosterols and other types. We then used SwissTargetPrediction to predict potential targets of the drugs. Based on a species of *Homo sapiens* and a probability >0.2, we ultimately obtained 125 potential drug targets and their corresponding 28 potential active ingredients after deduplication. There were 2 targets (7%) of Fritillariae Thunbrgii Bulbus, 3 (11%) of Coicis Semen, 2 (7%) of Panax Notoginseng, 19 (67%) of Resina Draconis, 1 (4%) of Panax Notoginseng and Resina Draconis, and 1 (4%) of Panax Notoginseng and Fritillariae Thunbrgii Bulbus.

**Figure 2:**
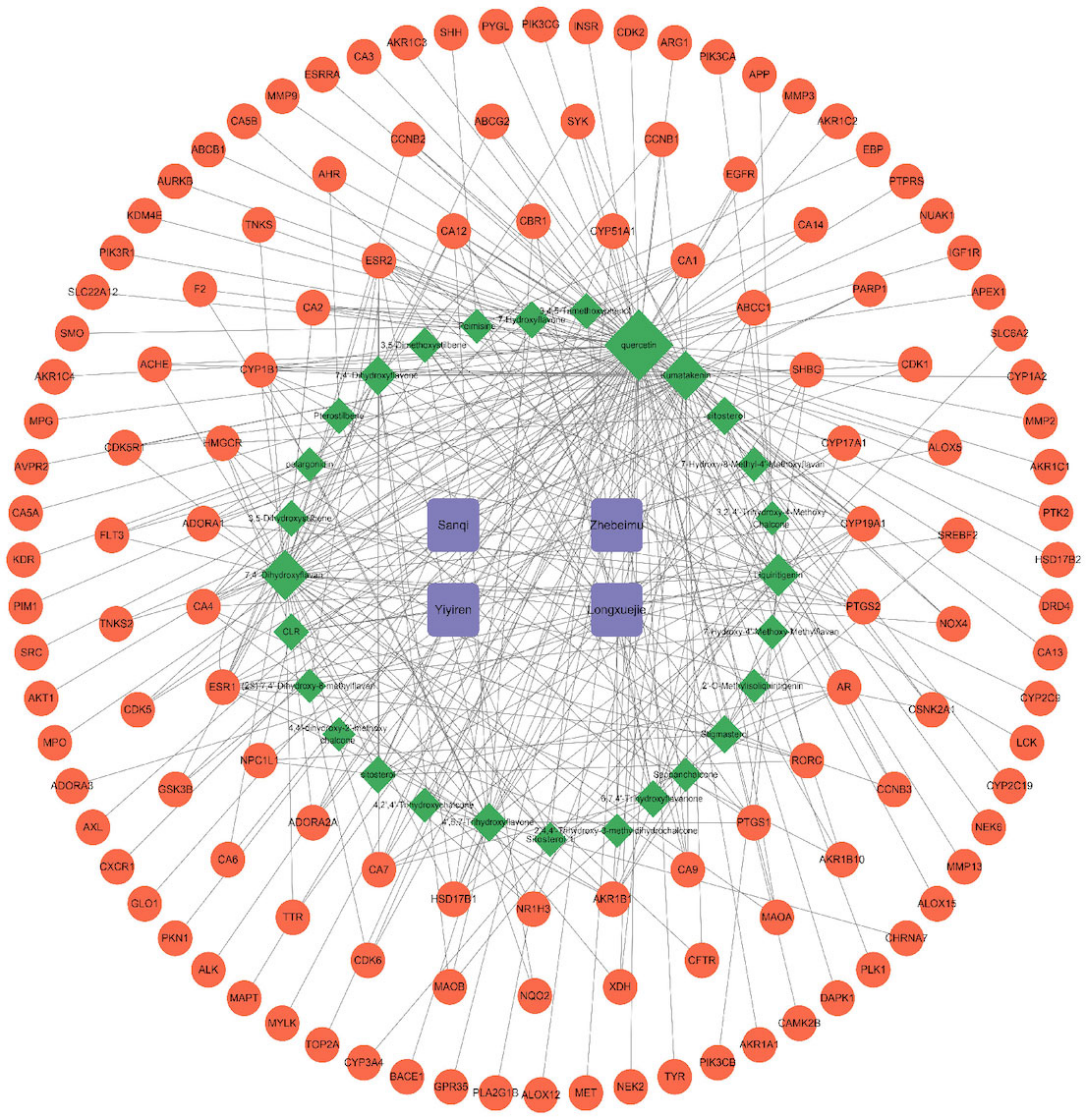
Hern-Compound-Target Network of SZC. The purple rectangular nodes represent the herb of SZC; the green diamond nodes represent the potential active compunds, the nodes were visualized in degree value, the larger the node, the higher the degree value; The red circular nodes represent the targets; The edges represent the relationship between them.

The 28 potential active ingredients and 125 related targets were used to construct a compound–target network and screen key active ingredients. The network included a total of 161 nodes and 353 edges (**Figure 2**). As shown in **Table 1**, we screened out 10 key active ingredients based on the degree value in the network, the higher the degree value of the active ingredient was, the more likely it was to have therapeutic efficacy.

**Table 1:** Potential active compounds information of SZC Network.

Compound	Degree	Betweenness	Closeness Centrality	Herb
Quercetin	94	0.671052244	0.573476703	Panax Notoginseng
7,4’-Dihydroxyflavan	42	0.094965534	0.413436693	Resina Draconis
Kumatakenin	40	0.094965534	0.413436693	Resina Draconis
Liquiritigenin	23	0.112339312	0.381861575	Panax Notoginseng/Resina Draconis
7,4’-Dihydroxyflavone	15	0.040619543	0.367816092	Resina Draconis
4’,5,7-Trihydroxyflavone	14	0.019720761	0.366132723	Resina Draconis
β-sitosterol	12	0.094861501	0.351648352	Panax Notoginseng/Fritillariae Thunbrgii Bulbus
Stigmasterol	12	0.051924017	0.351648352	Panax Notoginseng
sitosterol	10	0.034606991	0.34261242	Coicis Semen
7-Hydroxyflavone	10	0.006013499	0.357941834	Resina Draconis

### 3.2 Therapeutic target prediction analysis

After searching the GeneCards, OMIM and Drug bank databases, we obtained 932 potential EMs-related disease targets after deduplication.

**Figure 3:**
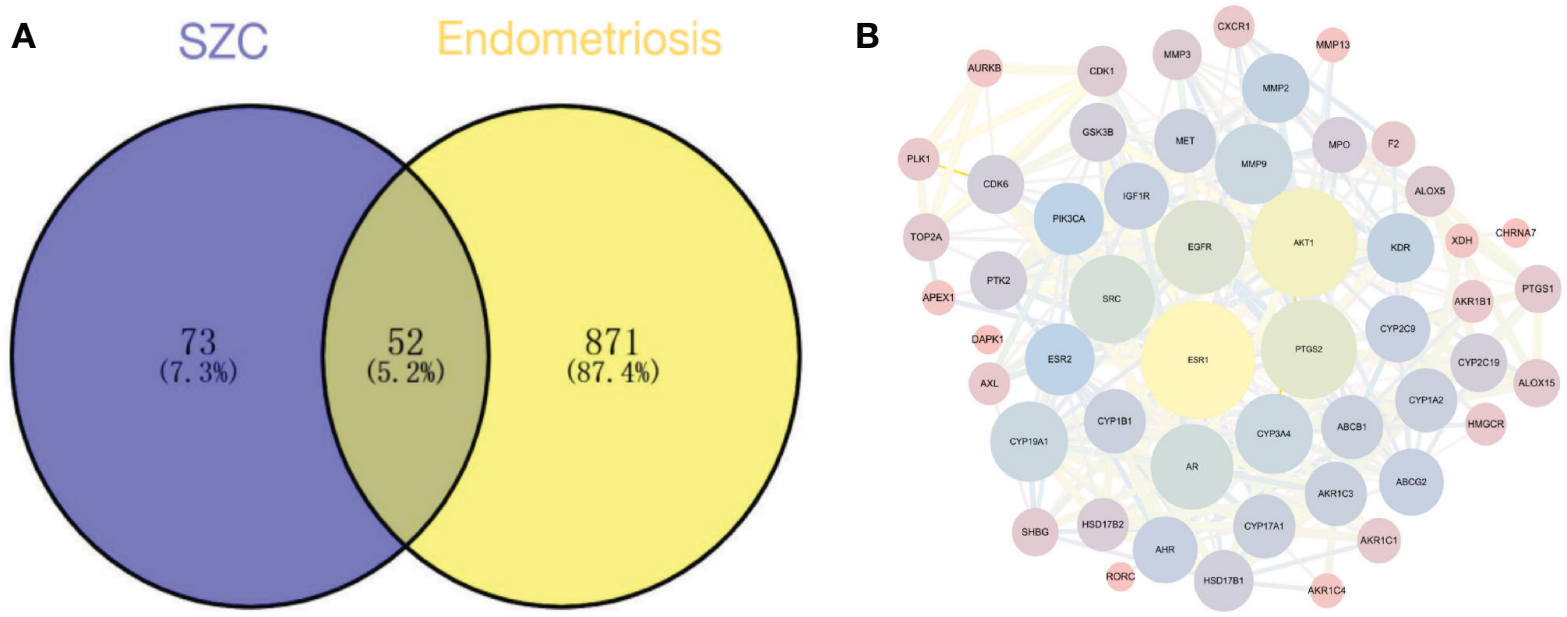
Potential therapeutic targets prediction. **(A)** The venn diagram of the overlapping target of SZC (Sanjie Zhentong Capsule) and Ems (Endometriosis). **(B)** The Protein-Protein Interaction (PPI) Network of SZC and Ems overlapping targets. The nodes in the figure represent the degree value, and the edges represent the interaction between the targets. The larger the node in the network, the greater the possibility of the target participating in the SZC treatment of Ems, and the thicker the line, the stronger the interaction between the targets.

We plotted a Venn diagram of SZC-related targets and EMs targets to identify 52 intersecting targets as potential targets of SZC in the treatment of EMs (**Figure 3** and **Supplementary Table S4**). We constructed a PPI network and topologically analyzed and visualized it, as shown in **Figure 3** and **Supplementary Table S5**. The PPI network contained a total of 52 nodes and 318 edges. We screened the following 9 targets with degree value ≥ 20 and betweenness centrality > 0.02 as key therapeutic targets (**Table 2**): ESR1, AKT1, PTGS2, EGFR, SRC, AR, matrix metallopeptidase 9 (MMP9), CYP19A1, and CYP3A4.

**Table 2:** Key targets of SZC in the treatment of Ems.

No.	Target	Degree	Betweenness Centrality	UniProt ID
1	ESR1	34	0.181977147	6VIG
2	AKT1	31	0.144313917	1UNR
3	PTGS2	27	0.072211573	5F19
4	EGFR	25	0.059824654	5HG8
5	SRC	23	0.027842759	1FMK
6	AR	22	0.036524645	5V8Q
7	MMP9	20	0.027798608	6ESM
8	CYP19A1	20	0.024684854	3S79
9	CYP3A4	20	0.036356048	5VCC

### 3.3 GO & KEGG pathway enrichment analyses

GO and KEGG pathway enrichment analyses were performed with 52 genes obtained from the PPI network. The top 10 molecular functions (MF), biological processes (BP) and cellular components (CC) terms identified in the GO enrichment analysis and the top 20 pathways among the KEGG enrichment pathways were screened and analyzed to explore the possible mechanisms by which SZC exerts therapeutic effects on EMs.

#### 3.3.1 GO enrichment analysis

**Figure 4:**
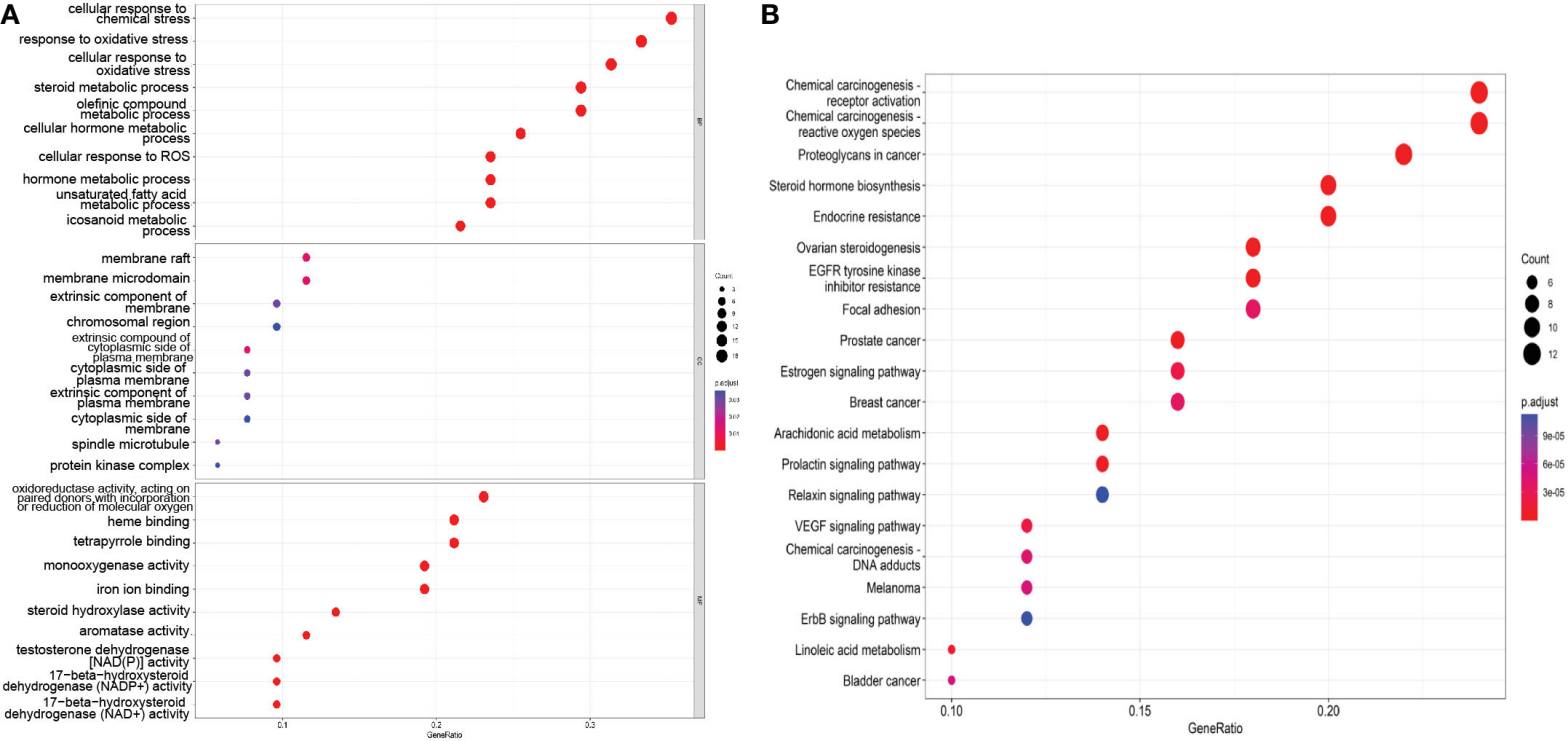
The GO and KEGG pathway enrichment analysis. **(A)** The top 10 GO terms of the potential therapeutic targets of SZC. The abscissa is GeneRatio, the larger the dot in the figure, the more genes involved. BP, biology process; CC, cytological component; MF, molecular function. **(B)** The top 20 KEGG pathway of the potential therapeutic targets of SZC. The abscissa in GeneRatio, the larger the dot in the figure, the more genes involved.

As shown in **Figure 4A**, the enriched BP terms included mainly the following: the cellular response to chemical stress, response to oxidative stress, cellular response to oxidative stress, steroid metabolic process, olefinic compound metabolic process, cellular hormone metabolic process, cellular response to ROS, hormone metabolic process, unsaturated fatty acid metabolic process, and icosanoid metabolic process. The enriched MF terms included mainly the following: oxidoreductase activity, acting on paired donors, with incorporation or reduction of molecular oxygen, heme binding, tetrapyrrole binding, monooxygenase activity, iron ion binding, steroid hydroxylase activity, aromatase activity testosterone dehydrogenase [NAD(P)] activity, 17−beta−hydroxysteroid dehydrogenase (NADP+) activity and 17−beta−hydroxysteroid dehydrogenase (NAD+) activity. The enriched CC terms included mainly the following: membrane raft, membrane microdomain, extrinsic component of membrane, chromosomal region, extrinsic compound of cytoplasmic side of plasma membrane, cytoplasmic side of plasma membrane, extrinsic component of plasma membrane, cytoplasmic side of membrane, spindle microtubule, protein kinase complex. These findings indicate that SZC treatment may affect these BPs, MFs and CCs.

#### 3.3.2 KEGG pathway enrichment analysis

We screened the top 20 pathways based on the gene ratios in the KEGG enrichment results, as shown in **Figure 4B**. The top 10 pathways were as follows: Chemical carcinogenesis-receptor activation, chemical carcinogenesis—ROS, proteoglycans in cancer, steroid hormone biosynthesis, endocrine resistance, ovarian steroidogenesis, EGFR tyrosine kinase inhibitor resistance, focal adhesion, the estrogen signaling pathway, and breast cancer.

### 3.4 Verification of the results by molecular docking

**Figure 5:**
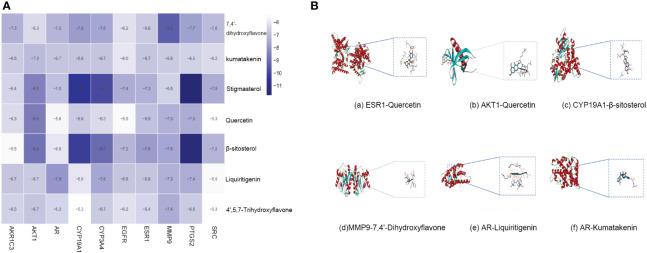
The molecular docking. **(A)** The binding energy of key compounds and targets (kcal/mol). **(B)** molecular docking results of high binding energy of SZC.

Molecular docking was performed with the top 10 potential therapeutic targets based on the degree value and betweenness centrality for 7 active ingredients by AutoDock 1.5.6. The results showed that all had moderate binding potential (**Figure 5A**), indicating that the mechanism by which SZC treats EMs is highly likely to involve binding of these key targets. **Figure 5B** shows images of the optimal docking of receptors and ligands with high binding energy after visualization.

### 3.5 SZC regulates cell proliferation and ROS production leading to apoptosis of EESCs

**Figure 6:**
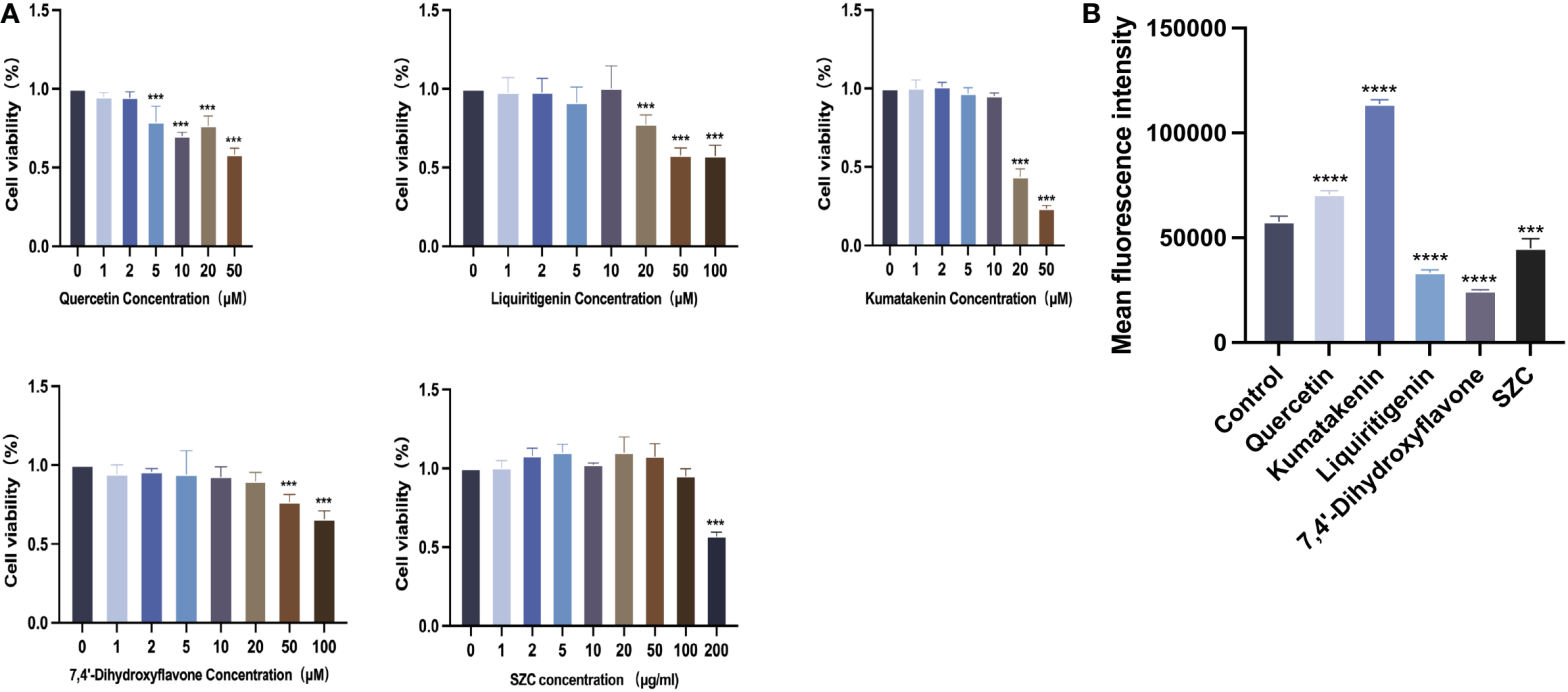
SZC and its active components inhibit proliferation of EESCs and promote ROS production of EESCs. **(A)** Cell viability effects of SZC and its active compounds treatment on EESCs. **(B)** Analysis of ROS production after SZC treatment by flow cytometry. The analysis of control group and SZC and its compounds group was assessed by DCFH-DA using flow cytometry. All data was presented as mean ± standard deviation, ***:p<0.001, compared with control group, ****:p<0.0001,compared with control group.

To determine whether SZC can inhibit EESC growth, promote apoptosis and regulate ROS production, we used SZC and 4 active ingredients of SZC to treat EESCs and detected cell viability with a CCK-8 kit. As shown in **Figure 6A** and **Supplementary Table S6**, all drug-treated groups lower viability than the control group; both quercetin at 5 µM and kumatakenin and liquiritigenin at 20 µM had a significant inhibitory effect on EESCs. SZC as a whole achieved a therapeutic effect by inhibiting EESCs proliferation, and 7,4’-dihydroxyflavone had an inhibitory effect on EESCs at 50 and 100 µM, but it has been reported in the literature that cytotoxicity occurs cells when the active ingredient concentration exceeds 20 µM (24). Based on the experimental data, when the concentration of 7,4’-dihydroxyflavone was less than or equal to 20 µM, there was no obvious inhibitory effect on ESCCs.

**Figure 7:**
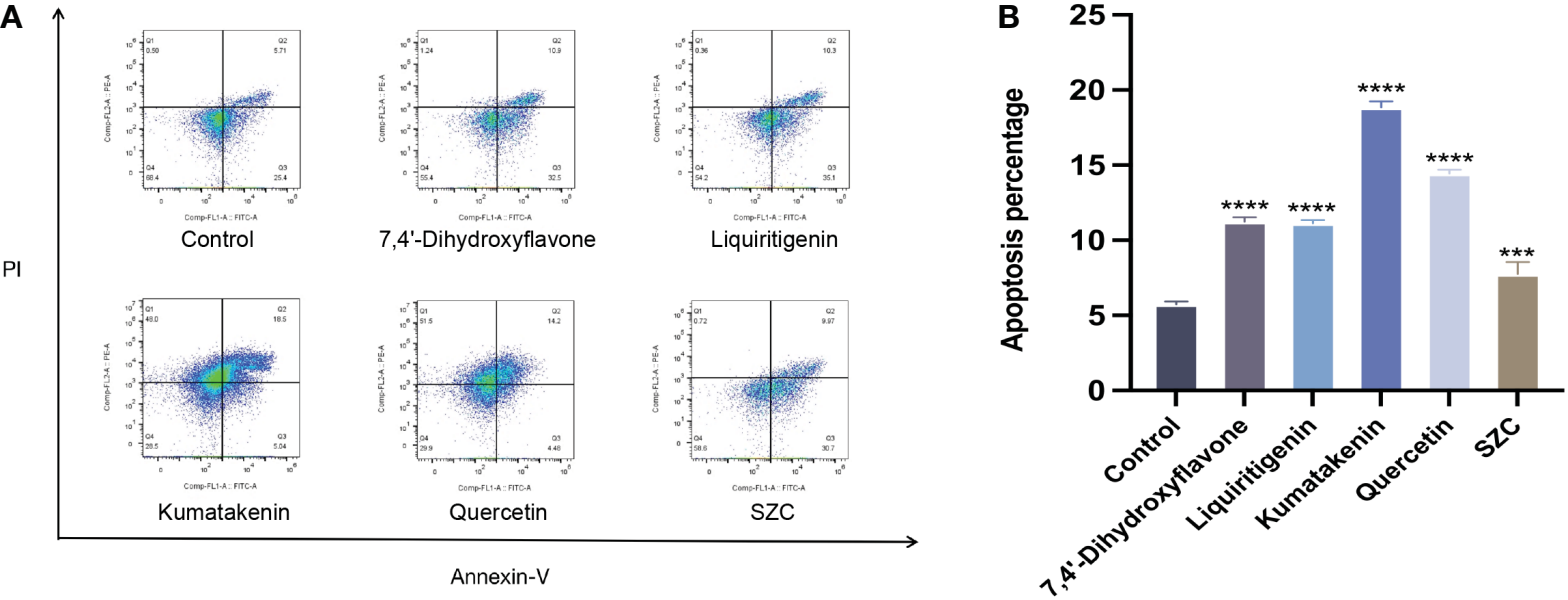
SZC and its active components promote apoptosis of EESCs. **(A, B)** Analysis of EESCs apoptosis after SZC treatment by flow cytometry. The analysis of control group and SZC and its compounds group was assessed by Annexin V/PI staining using flow cytomentry. All data was presented as mean ± standard deviation, ***:p<0.001,compared with control group, ****:p<.0001,compared with control group.

Subsequently, we investigated whether SZC and its components can promote the apoptosis of EESCs (**Figure 7**). In addition, quercetin and kumatakenin can also promote the production of ROS, while liquiritigenin, 7,4’-dihydroxyflavone and SZC inhibited the production of ROS (**Figure 6B**).

### 3.6 SZC treats EMs by regulating steroid synthesis and cell proliferation-associated mRNA expression

**Figure 8:**
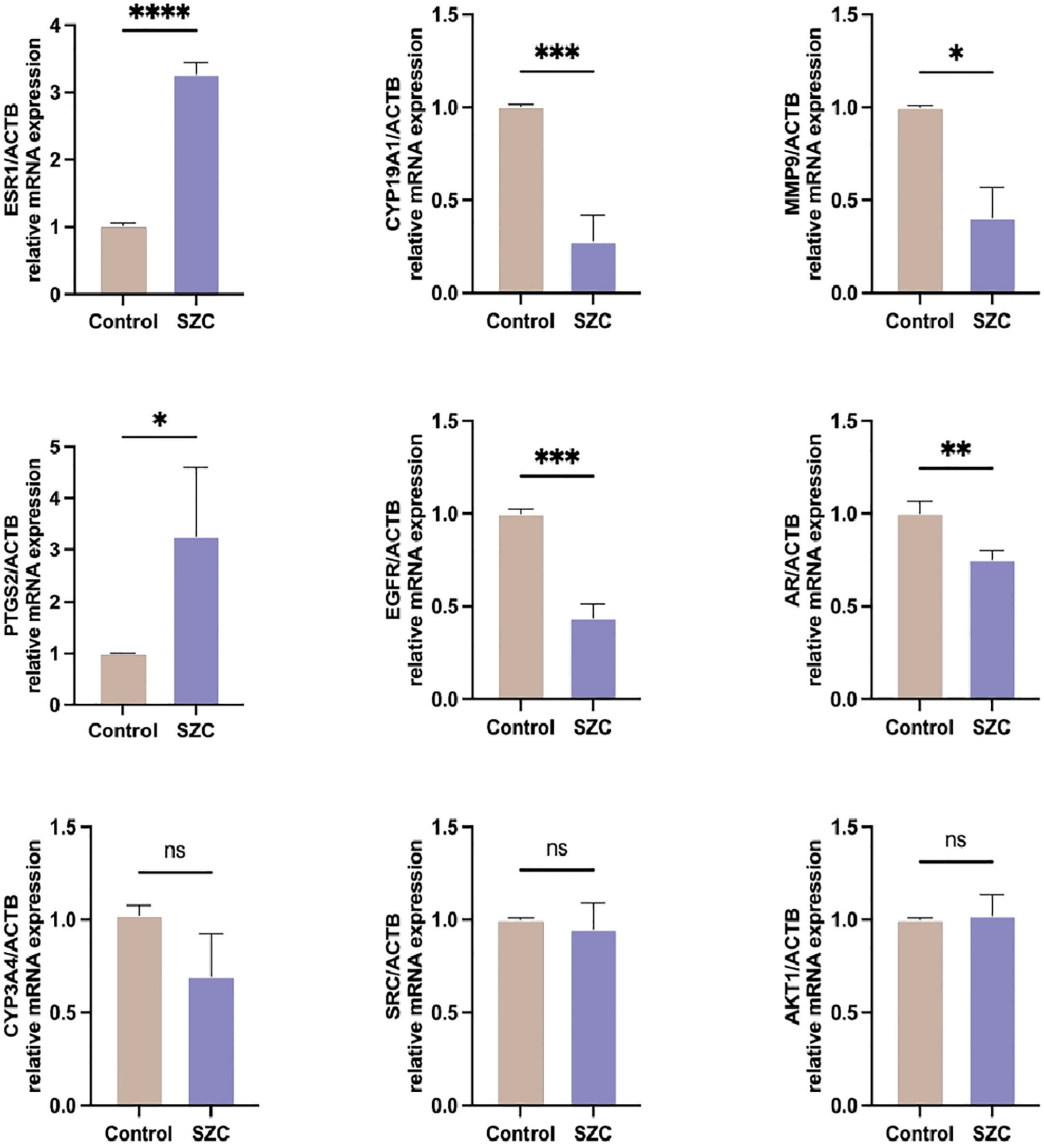
Analysis of predicted target genes mRNA expression after SZC treatment by RT-PCR. Data are presented as means ± standard deviation of three independent replicates. *P<0.05, **P<0.01, ***P<0.001, ****P<0.0001, and n.s P≥0.05, compared with the control group.

To verify whether the SZC acts on the EMs through the targets predicted by the previous network pharmacology experiment, we compared the expression of related genes *via* RT−PCR. ESR1 is an estrogen receptor and ligand-activated transcription factor that plays a key role in endometriosis (25). CYP19A1 and CYP3A4 are both members of the CYP superfamily of enzymes, CYP19A1 catalyzes the last steps of estrogen biosynthesis (26), while CYP3A4 is involved in the local degradation and homeostasis of steroid hormones and can promote endometriotic cell proliferation (27). AR is a steroid hormone-activated transcription factor involved in regulating steroid hormone biosynthesis. EGFR is a cell surface protein that binds to epidermal growth factor, thus inducing receptor dimerization and tyrosine autophosphorylation and leading to cell proliferation (28). MMP9 is a zinc-dependent endopeptidases involved in the breakdown of the extracellular matrix in normal physiological processes, and it has been reported that it may be involved in the proliferation, invasion, and migration of EESCs (29). AKT1, as a member of the human AKT serine-threonine protein kinase family, can regulate many processes, including metabolism, proliferation, cell survival, growth and angiogenesis (30). SRC is a nonreceptor protein tyrosine kinase that participates in signaling pathways that control a diverse spectrum of biological activities, including gene transcription, immune response, cell adhesion, cell cycle progression, apoptosis, migration, and transformation (31). As shown in **Figure 8**, SZC inhibited the expression of the CYP19A1, EGFR and MMP9 genes and promoted the expression of the ESR1 and PTGS2 genes. It did not significantly affect AKT1, CYP3A4 and SRC gene expression. However, the expression of CYP3A4 showed an overall downward trend.

## 4 Discussion

SZC is a TCM compound known for its ability to soften and reduce knots (10, 11, 24), promote menstrual flow and relieve pain (9, 15). SZC has a good curative effect in the treatment of EMs (12–14), so its clinical use has gradually begun to increase. Several studies have shown that SZC can shrink lesions in rats with EMs, inhibit multiple cytokines, inhibit inflammation and angiogenesis, and promote EESC apoptosis (11, 17, 22). In addition, our previous study has demonstrated that SZC is a more effective pain reliever than GnRH-α or OCs and that it improves quality of life (QoL) with fewer adverse events in patients with moderate to severe EMs (16).

However, it is well known that the therapeutic properties of TCM compounds are mediated through multiple components, targets, and pathways during disease treatment. The effects of SZC on EMs and the underlying mechanisms have not been systematically evaluated. This study aimed to explore the potential mechanism of SZC in the treatment of EMs using network pharmacology and to verify this mechanism through molecular docking and *in vitro* experiments.

We screened a total of 28 SZC-related active ingredients by consulting the TCMSP database and related literature. The active ingredients mainly include flavonoids and plant sterols, such as quercetin, 7,4’-dihydroxyflavone, kumatakenin, liquiritigenin, stigmasterol, and 7,4’-dihydroxyflavan. Flavonoids such as quercetin and some other active ingredients have been shown to be effective against EMs (32). In addition, a study confirmed that quercetin can inhibit cell proliferation and induce EESC apoptosis by significantly reducing CCND1 gene expression, and this process is related to mitochondrial membrane dysfunction and ROS production (33). In addition, quercetin can inhibit the expression of ERα, ERβ, and PR in the hypothalamus, pituitary gland and endometrium by regulating the hypothalamic–pituitary–gonadal axis and promote the binding of estrogen and progesterone to their receptors, thereby causing ectopic endometrial atrophy (34). Kumatakenin shows anticancer activity by inducing the apoptosis of ovarian cancer cells and inhibiting the alternative activation of tumor-associated macrophages (35). Liquiritigenin, a natural ERβ agonist that has good antioxidant and anti-inflammatory effects, can significantly inhibit ovarian cancer cell viability, reduce migration and invasion, and promote apoptosis (36, 37). Liquiritigenin may play a role in the treatment process through the estrogen signaling pathway. Resveratrol is currently the most studied TCM ingredient for the treatment of EMs, and it can effectively delay the progression of EMs and improve related symptoms (32, 38–40). Although it is a component of Resina Draconis, resveratrol was not included in this study due to its relatively low bioavailability. Studies have shown that steroids such as stigmasterol and β-sitosterol have antioxidant properties, inhibit the proliferation of various tumor cells, have anti-inflammatory and analgesic effects, and inhibit the progression of EMs (41–45).

From PPI network analysis, we obtained a total of 52 potential targets. After topological analysis and visualization of the network, we suggest that ESR1, AKT1, PTGS2, EGFR, SRC, AR, MMP9, CYP19A1 and CYP3A4 are key targets for treatment.

EMs is estrogen-dependent and closely related to steroid metabolism, so we hypothesized that expression of the ESR genes may play an important role in hormonal regulation. In EMs, the expression of ERβ is upregulated, while the expression of ERα is decreased, resulting in increased local estradiol (E2) levels in the endometrium, a pathological feature of EMs (46). Previous studies have demonstrated that ESR1 mRNA expression is attenuated and that ESR2 mRNA expression is significantly increased in EESCs compared with normal endometrial stromal cells (47). Therefore, ectopic endometrial tissue inhibits the expression of ERα by overexpressing ERβ, resulting in reductions in ERα-mediated progesterone receptors and increasing progesterone resistance in patients with EMs, which may be related to infertility (25).

The AR gene, located on the X chromosome, is associated with the hormonal regulation of EMs (48, 49), and studies have confirmed that endometriotic cysts are monoclonal in origin and are associated with AR (49). Androgen stimulates endometrial cell proliferation (50). In addition, estrogen induces the mRNA and protein expression of AR in the endometrium, allowing androgens to exert biological effects on the uterus through AR (28).

The estrogen-metabolizing activities of CYP enzymes have been implicated in EMs (51). CYP19A1 and CYP3A4 are members of the CYP superfamily of enzymes (26). CYP19A1 is the main component of the aromatase enzyme and plays an important role in estrogen biosynthesis, and its expression is significantly increased in EMs. In addition, the proinflammatory factor PGE2 has been shown to induce the expression of CYP19A1 in EMs (52, 53). CYP3A4, which plays an important role in the local degradation and homeostasis of steroid hormones, can promote endometriotic cell proliferation (27, 54–56). Furthermore, studies have confirmed that IGF-1 mRNA expression in EESCs is significantly higher than that in normal endometrial cells, and stimulates ESR2 and CYP19A1 mRNA expression (57).

The MMPs are a family of zinc-dependent endopeptidases known to regulate the migration, invasion and proliferation of various cell types (29). As a member of this family, MMP9 is expressed at high levels in EESCs and may play a key role in the occurrence and progression of EMs by inhibiting the migration of EESCs (58–61).

Studies have shown that EGFR, a tyrosine kinase receptor, can promote the differentiation and proliferation of EESCs (62, 63). Furthermore, EGFR may be involved in disease progression in advanced EMs, promoting EESC proliferation and adhesion by increasing MMP-7 levels (64).

AKT is a serine/threonine kinase implicated in cell cycle control, apoptosis evasion, cell proliferation, and metabolic processes (30). Studies have shown that the PI3K/Akt signaling pathway is abnormally activated in EMs, and AKT1 is highly expressed in the ectopic endometrium (65–67). Increased AKT1 phosphorylation may be involved in the coordination of apoptosis/proliferation in endometriotic cells (68).

Previous research has suggested that EMs may be an epigenetic disease related to the PTGS2 gene, and genetic polymorphisms in PTGS2 are associated with a high risk of EMs (69, 70). PTGS2 mRNA expression in the endometrial and ovarian lesions of EMs patients is significantly correlated with serum CA-125 and the diameter of endometriomas (69). In addition, studies have shown that PTGS2 increases the risk of pain in women with EMs (71), and the cyclooxygenase II (COX-2) enzyme, encoded by the PTGS2 gene, is naturally induced by aromatase and is involved in the conversion of arachidonic acid to prostaglandins, which may induce dysmenorrhea (26). Decreased PTGS2/COX-2 expression may lead to decreased oocyte quality, which has been suggested as a possible mechanism of EMs-related infertility (72). A previous study has demonstrated that SZC can reduce serum COX-2 levels in patients with dysmenorrhea (73), but the RT−PCR results of this study revealed that PTGS2 mRNA expression was significantly elevated after SZC treatment. This results may have been related to its gene polymorphisms and regulation of diversity biological behaviors.

According to the results of GO and KEGG enrichment analyses, the mechanism of SZC in the treatment of EMs involves mainly the following potential biological processes and pathways: oxidative stress, steroid metabolism, apoptosis and proliferation.

Estrogen dependence and progesterone resistance are major endocrine features of EMs. Estrogen production and progesterone resistance in endometriotic lesions promote apoptosis and inflammation and reduce immune function (46, 74). One study has shown that ESR2 and CYP19A1 gene expression is increased in EMs patients, and that ESR1 gene expression is reduced, thereby promoting the local biosynthesis of E2 in EMs (52). E2 promotes inflammation through ESR2-induced COX-2- PGE2 signaling (70). In addition, studies have confirmed that E2 can promote the proliferation of ectopic endometrial cells through the IGF1-mediated PI3K/Akt signaling pathway (75–77). According to our network pharmacology research and GO and KEGG pathway enrichment analyses, it was found that hormone metabolism may play a key role in the treatment of EMs by SZC, and the relevant predicted targets were verified by RT−PCR, revealing that SZC can treat EMs by upregulating ESR1 mRNA expression and inhibiting AR and CYP19A1 mRNA expression in EESCs.

ROS are known as the main factors in EMs pathophysiology. Cell proliferation, damage and apoptosis are controlled by regulation of the levels of ROS. Studies have shown that the expression level of ROS-related indicators are higher, while the levels of antioxidant are lower, in ectopic endometrium tissue than in normal endometrium tissue (78). According to the network pharmacology results, we believe that the PI3K/Akt pathway may be involved in regulating ROS production. ROS are important byproducts of cell metabolism. Moderate levels of ROS can promote the proliferation and invasion of ectopic endometrial cells. However, accumulation of excess ROS causes damage to cellular components such as membranes, proteins, and DNA and induces autophagy to remove damaged cellular components, resulting in cell death (79). ROS can activate the PI3K/Akt pathway, which plays an important role in regulating autophagy, thereby regulating ROS levels and maintaining cell proliferation. Studies have shown that decreased expression levels of the AKT and SRC genes correspond to increased autophagy (80). In addition, inflammatory mediators and cytokines promote the flow of macrophages, which in turn accelerates the accumulation of intracellular ROS (81), activating the PTGS2 gene and thereby inhibiting apoptosis through the NF-κB signaling pathway (82).

When the levels of ROS are appropriately increased, some natural flavonoids can inhibit AKT activation, promote Beclin-1 expression, increase caspase-3 activity, and then promote apoptosis (83, 84). In addition, β-sitosterol is a potential antioxidant that protects cells from lipid peroxidation and ROS-mediated damage (85). Through the network pharmacology and experimental verification, we found that quercetin and kumatakenin increased the levels of ROS, while liquiritigenin and 7,4’-dihydroxyflavone decreased the levels of ROS. Overall, SZC achieved a therapeutic effect by inhibiting oxidative stress. Considering the dual effects of ROS, we believe that liquiritigenin and 7,4’-dihydroxyflavone may alleviate EMs by decreasing the production of ROS. A previous study has suggested that quercetin can disrupt mitochondrial function and increase ROS production by inhibiting the MAPK and PI3K/AKT pathways, thereby inhibiting proliferation and promoting apoptosis (33). Kumatakenin and quercetin are both natural flavonoids that may act on EESCs through the same mechanism. However, further experiments are needed to verify whether SZC and its components affect EMs through the aforementioned mechanisms.

Apoptosis plays a crucial role in maintaining tissue homeostasis, a program that eliminates dysfunctional or excess cells. According to previous studies, inhibition of apoptosis and hyperproliferation of EESCs are the main reasons for the progression of EMs (78, 86). Based on the present study, we suggest that SZC may regulate cell proliferation and apoptosis in EMs by acting on genes such as EGFR, MMP9, CYP3A4, AKT1 and SRC. AKT1 has been confirmed to be abnormally activated in EESCs (87). This gene can activate a range of downstream factors *via* the PI3K/AKT/mTOR signaling pathway, such as EGFR and VEGF, to promote cell proliferation and inhibit apoptosis (88). Previous studies have found that EGF expression is significantly increased in patients with severe EMs (63, 64). SRC, CYP3A4 and MMP9 are all related to the proliferation of EESCs. In our experiments, we found that SZC inhibited EESC proliferation and promoted EESC apoptosis. In addition, we found that SZC significantly inhibited EGFR and MMP9 mRNA expression, but there was no significant difference in the AKT1, CYP3A4 and SRC mRNA expression. A review of the literature in combination with experimental data revealed that the concentration of the predicted active ingredient in SZC is far higher than the concentrations of the drugs used to inhibit EESCs in the experiment. Therefore, we believe that the potential active ingredient we screened reaches a concentration in patients who take SZC that can effectively inhibit EESCs. However, due to the multicomponent characteristic of TCM, research on the compounds in SZC is still improving, and further research is needed.

## 5 Conclusion

In this study, we explored the potential mechanism of SZC in the treatment of EMs through network pharmacology analysis, molecular docking, and *in vitro* experiments. We found that quercetin, kumatakenin, 7,4’-dihydroxyflavonoids, etc., may be the main active components of SZC, and we identified ESR1, AKT1, PTGS2, EGFR, SRC, AR, MMP9, CYP19A1, and CYP3A4 as key therapeutic targets. GO and KEGG pathway enrichment analyses of the therapeutic targets suggested that SZC exerts its therapeutic effects mainly by affecting oxidative stress, steroid metabolism, apoptosis and proliferation. Furthermore, we verified the good binding affinity between predicted potential key components and targets by molecular docking. Finally, our *in vitro* experiments verified that SZC can play a therapeutic role in EMs by regulating the expression of steroid hormone biosynthesis-related genes, inhibiting EESC proliferation and oxidative stress, and promoting apoptosis.

## Data availability statement

The original contributions presented in the study are included in the article/**Supplementary Material**. Further inquiries can be directed to the corresponding author.

## Ethics statement

The studies involving human participants were reviewed and approved by Human Research Ethics Committee of the Obstetrics and Gynecology Hospital, Fudan University. The patients/participants provided their written informed consent to participate in this study.

## Author contributions

LW and YW conceptualized the study. RG and ZY led the experimental design and wrote the manuscript. RG, ZY, and YW performed the experiments. LW and YW corrected the manuscript. All authors contributed to the article and approved the submitted version.
