# Supplementary figures and images for: Network pharmacology and experimental validation to explore the potential mechanism of Sanjie Zhentong Capsule in endometriosis treatment
Source: Front Endocrinol (Lausanne). 2023 Feb 3;14:1110995. doi: 10.3389/fendo.2023.1110995 (PMC9935822; doi:10.3389/fendo.2023.1110995)

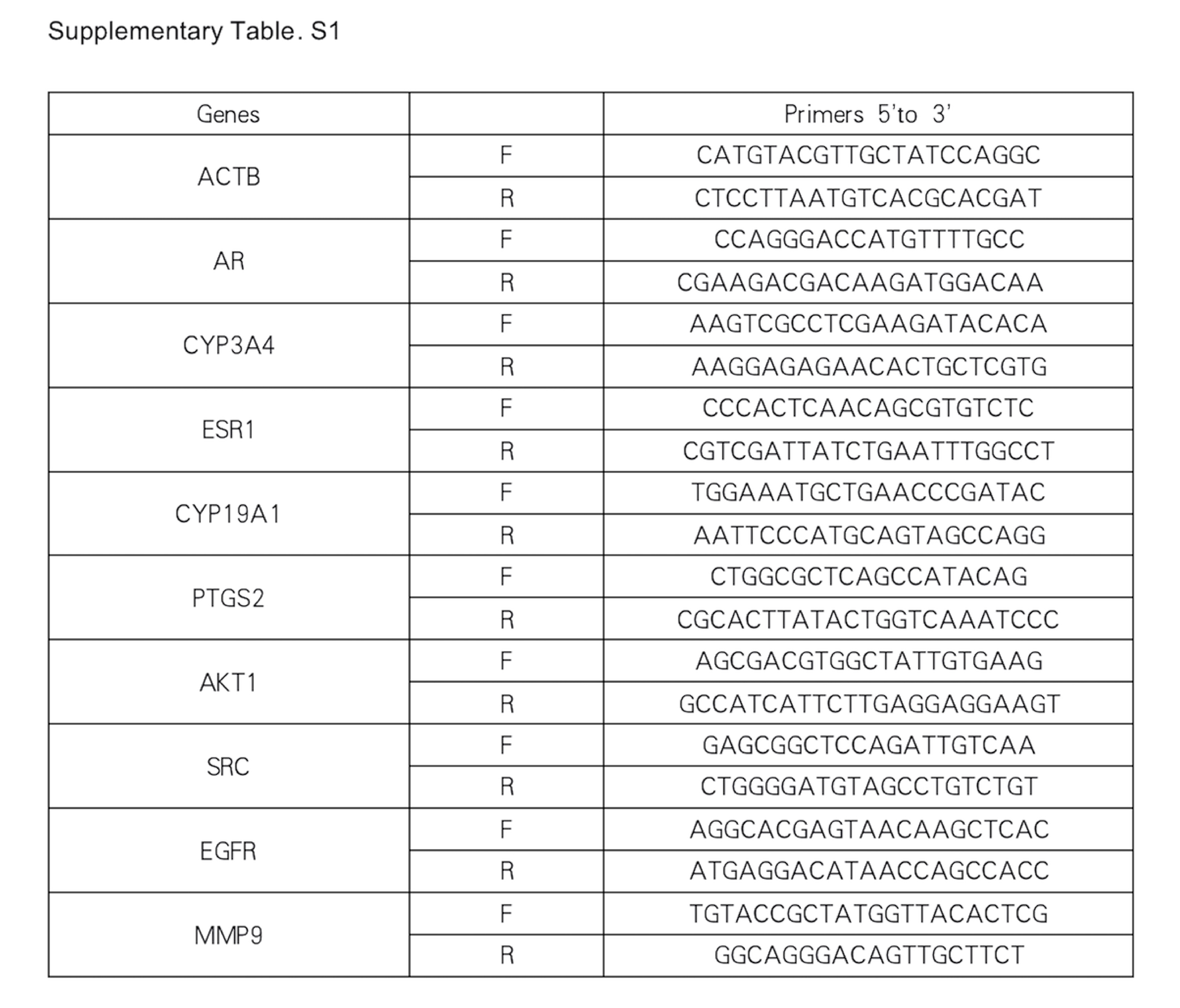

Supplement: Supplementary file 1 [file Image_1.jpeg]
